# Stillbirths in Cameroon: an analysis of the 1998–2011 demographic and health surveys

**DOI:** 10.1186/s12884-022-04993-5

**Published:** 2022-10-01

**Authors:** Adidja Amani, Jobert Richie Nansseu, Guy Ferdinand Ndeffo, Andreas Ateke Njoh, Fabrice Zobel Lekeumo Cheuyem, Paul Roger Libite, Athanase A. Ateba, Solange Ngo Bama, Martina Lukong Baye, Georges Nguefack-Tsague, Robinson Enow Mbu

**Affiliations:** 1 Department of Family Health, Ministry of Public Health, Yaounde, Cameroon; 2 Faculty of Medicine and Biomedical Sciences, University of Yaounde I, Yaounde, Cameroon; 3 Department of Disease, Epidemics and Pandemics Control, Ministry of Public Health, Yaounde, Cameroon; 4 Department of Public Health, Faculty of Medicine and Biomedical Sciences, University of Yaounde 1, Yaounde, Cameroon; 5 Ministry of Economy, Planning and Regional Development, National Institute of Statistics, Yaounde, Cameroon; 6 Faculty of Science, University of Buea, Buea, Cameroon; 7 Yaounde, Cameroon

**Keywords:** Stillbirth, Prevalence, Risk factor, Cameroon

## Abstract

**Background:**

Many countries, including Cameroon, have found it challenging to estimate stillbirths, as there are limited available reports accurately. This analysis aimed to assess stillbirth rates and identify risk factors for stillbirth in Cameroon using successive Demographic and Health Survey data.

**Methods:**

We conducted a cross-sectional analysis of data collected during the Demographic and Health Surveys of 1998, 2004, and 2011. Data were analyzed using SPSS 20.0. Logistic regressions were used to identify factors associated with stillbirth through odds ratios (ORs) at 95% confidence intervals (CI). Results were considered statistically significant at *p*-value less than 0.05.

**Results:**

The crude stillbirth rate was 21.4 per 1,000 births in 2004 and 24 per 1,000 births in 2011, with respective standard errors of 1.8 and 1.3. The stillbirth rate increased with the mother's age (*p* < 0.001). The stillbirth rate reduction was prolonged between 1998 and 2011, with an annual reduction rate of 1.6%. The study observed that residing in rural areas, low socioeconomic status, and low level of education were risk factors associated with stillbirths.

**Conclusion:**

Cameroon's stillbirth rate remains very high, with a slow reduction rate over the last 20 years. Although some efforts are ongoing, there is still a long way forward to bend the curve for stillbirths in Cameroon; supplementary strategies must be designed and implemented, especially among rural women, the poor, and the less educated.

## Background

The World Health Organization defines a stillbirth, according to the 10^th^ revision of the International Classification of Diseases (ICD-10), as a baby born with no signs of life weighing ≥ 1000 g or after at least 28 completed weeks of gestation [1]. An updated national, regional, and worldwide estimate of stillbirths was published in 2020, which estimated that close to two million stillbirths occurred in 2019, with an estimated average stillbirth rate of 13.9 per 1000 births globally, with the worst values in Sub Sahara Africa [2]. The major causes of stillbirths include childbirth complications, maternal infections in pregnancy such as syphilis or malaria, maternal conditions, among which hypertension and diabetes, fetal growth restriction, and congenital abnormalities [3, 4]. Advanced maternal age, socioeconomic factors, nutritional factors, prior stillbirths, pregnancy complications and lifestyle factors constitute the major risk factors for stillbirths in developing countries [3].

Like other developing countries, Cameroon is committed to stillbirth reduction under the Global Strategy for Women's Child and Adolescent health target of decreasing the stillbirth rate to ≤ 12 per 1,000 births by 2030 [4]. However, the information gap concerning stillbirths is still immense. Previous summaries of stillbirth estimates attributed the stillbirth rate in Cameroon to 20 per 1,000 births in 2004, 25.6 per 1,000 births in 2009 [5], and 19.6 per 1,000 births in 2015 [2]. However, no stillbirth rate input data from Cameroon was included in any of these estimation exercises, as the estimate for Cameroon was essentially derived from a covariate-based modeling approach.

Nationally representative surveys such as the Demographic and Health Surveys (DHS) provide essential information on child health and mortality; many of these surveys also include pregnancy and birth estimates. However, there is evidence that in some settings, these surveys under-captured adverse birth outcomes, especially stillbirths. This information can nevertheless help in providing some guidance on national stillbirth rates. The stillbirth data has never been previously analyzed in the DHS reports successively conducted in Cameroon in 1998, 2004, and 2011 [5–7]. These surveys did not single out stillbirths and instead used the term "wasted pregnancies," an aggregate of three pregnancy outcomes, including induced abortion, miscarriage, and stillbirths. Hence, to the very best of our knowledge, this is the first-time stillbirth data are being looked at. In addition, although understanding risk factors for stillbirths in women is beneficial both for child survival and reduction in maternal mortality [6], investigating potential risk factors for stillbirths in Cameroon has received very little attention until now [7, 8].

Therefore, there is a crucial gap in terms of both accurate national prevalence estimates and risk factors for stillbirths in Cameroon. Hence, there is an urgent need to improve the understanding of stillbirths and recommend targeted interventions throughout the country. This study aims to determine the prevalence and risk factors for stillbirths in Cameroon and the evolution from 1998 to 2011.

## Methods

### Study setting

Cameroon is a country in Central Africa with an estimated population in 2022 of over 27.6 million [9, 10]. The country has ten administrative regions; it has two capital cities: Yaoundé, the political capital, and Douala, the economic capital. The population is very young: 43.6% of Cameroonians are less than 15 years [10].

### Study design and participants

We conducted a cross-sectional study. Data was collected during the DHS in 1998, 2004, and 2011 [11, 12]. The DHS is a nationally representative household survey that provides data for a wide range of monitoring and impact evaluation indicators in population, health, and nutrition. It collects information on households, women aged 15–49, men aged 15–59, and children. The seventh phase of the DHS improved the way to collect data on miscarriage, abortion, and stillbirth by collecting the dates those events occurred during the previous five years. During the earlier phases, the information about those events generally covered the period between the beginnings of a woman's fertility until the interviewer visited the household. This way of collecting data does not allow estimating the stillbirth rate by year but according to the period mentioned above. This situation could cause bias in estimating the stillbirth rate.

The data source also provides information on the contextual characteristics of these women and thus offers the opportunity to examine risk factors. This study concentrated on women aged 15–49 years and defined stillbirths as late fetal deaths occurring at seven months of gestation [1].

### Data collection

The DHS used an individual questionnaire administered to all women aged 15–49 years in selected households. This questionnaire collected, among other variables, data on the characteristics of women (age, educational level, partner's level of education, marital status, setting (urban vs. rural), region of residence, and wealth index quintile), their birth history and their complete pregnancy histories (miscarriage, abortion, stillbirths). The latter information was gathered by asking questions about the total number of miscarriages, abortions, and stillbirths from the beginning of the woman's fertile period to the date of data collection in the household. {Until the fifth phase of DHS, data on birthday history, miscarriages, abortions, and stillbirths were collected over the abovementioned period. Nevertheless, a particular focus was given to the last miscarriage, abortion, or stillbirth over the previous five years or the beginning of the fertile period to the interview date.}

### Statistical analysis

Data were analyzed using SPSS 20.0 (SPSS IBM Inc., Chicago, Illinois, USA). The respondent characteristics are presented as weighted numbers, frequencies, and percentages. The stillbirth rate was calculated as the total number of babies reported by their mothers as stillborn divided by the total number of births (live and stillborn) per 1,000 total births reported. Data is weighted when calculating the stillbirth rate to obtain robust estimates. These estimates covered an extensive period, from the beginning of women's fertile periods to the survey's completion date.

Additionally, we estimated the Average Annual Rate of Reduction (AARR) of stillbirths by quantifying the rate of change in the prevalence from 1998 to 2011, with a change in rate assumed to take an exponential progression. For any given year t, if the prevalence was known to be Yt, and the annual rate of reduction was constantly b%, then the prevalence of the following year, denoted as Yt + 1, was calculated as Yt + 1 = Yt*(1-b%) [10]. The Chi-2 test was used to seek associations between categorical variables. Univariable and multivariable logistic regression analyses were used to identify factors associated with stillbirth occurrence through odds ratios (ORs) and their 95% confidence intervals (CIs). Results were considered statistically significant for a p-value less than 0.05.

### Ethical considerations

The Ethical Review Board of the Faculty of Medicine and Biomedical Sciences of the University of Yaounde 1 approved the study, in addition, we received administrative authorization from the General Manager of the Cameroon National Institute of Statistics. We could not obtain participant approvals in our retrospective analyses, though this was obtained before DHS data were collected. All methods were performed following the relevant guidelines and regulations. We scrupulously respected the anonymity and confidentiality of the information collected.

## Results

### Characteristics of the study population

In total, 5 501, 10 656, and 15 426 women were included in our 1998, 2004, and 2011 analyses. Women aged 15–19 were the most represented (23–25%). The study by place of residence showed that the Littoral region (excluding Douala) in 2004 (17.4%) was the most represented, followed by the Centre (excluding Yaounde) region in 2011 (16.8%). Women in rural areas comprised about half of the study sample (Table 1). Around 18–24% had no education, which was quite similar among their partners. Most women were married (47–53%) or single (26–28%). According to the wealth index, the very poor and poor quintiles represented 17.6% and 17.1% of the sample in 2004 and 14.9% and 19.8% in 2011, respectively (Table 1).

**Table 1:** Univariate logistic regression

	**1998**		**2004**		**2011**	
**Variables**	**OR (95% CI)**	**P**	**OR (95% CI)**^**a**^	**P**	**OR (95% CI)**^**a**^	**P**
**Age group**		** < 0.001**		** < 0.001**		** < 0.001**
15–19	1		1		1	
20–24	6, 43 (247; 16, 70)		5.72 (2.97; 11.00)		4.16 (2.53; 6.85)	
25–29	11, 68 (4.59; 29, 72)		9.73 (5.13; 18.46)		6.32 (3.88; 10.28)	
30–34	15, 44 (6, 06; 39, 30)		13.45 (7.10; 25. 47)		10.50 (6.49; 16.99)	
35–39	23, 64 (9.39; 59, 49)		17.38 (9.18; 32.90)		14.03 (8.71; 22.59)	
40–44	27, 66 (10.91; 70, 15)		19.19 (10.06; 36.61)		16.53 (10.21; 26.76)	
45–49	44, 39 (17.53; 112, 41)		22.03 (11.53; 42.09)		15.71 (9.66; 25.55)	
**Region of residence**				** < 0.001**		** < 0.001**
Adamaoua			1		1	
Centre			1.53 (0.94; 2.48)		0.57 (0.38; 0.84)	
Douala			0.48 (0.26; 0.88)		0.49 (0.33; 0.72)	
East			2.08 (1.29; 3.36)		0.69 (0.46; 1.03)	
Far-North			1.51 (0.94; 2.42)		1.20 (0.89; 1.63)	
Littoral			1.37 (0.83; 2.26)		0.61 (0.40; 0.94)	
North			0.39 (0.20; 0.75)		0.87 (0.63; 1.21)	
North-West			0.76 (0.43; 1.34)		0.34 (0.22; 0.51)	
West			1.53 (0.96; 2.45)		0.69 (0.48; 0.99)	
South			0.96 (0.55; 1.68)		0.64 (0.42; 0.96)	
South-West			0.78 (0.44; 1.39)		0.53 (0.35; 0.80)	
Yaoundé			0.78 (0.45; 1.36)		0.52 (0.35; 0.76)	
**Type of residence**		** < 0.001**		** < 0.001**		** < 0.001**
Urban	1		1		1	
Rural	1.55 (1.20; 2.00)		1.67 (1.36; 2.06)		1.44 (1.23; 1.70)	
**Level of education**		** < 0.001**		** < 0.001**		** < 0.001**
No education	2.56 (1.82; 3.60)		1.76 (1.33; 2.33)		1.58 (1.27; 1.97)	
Primary	2.22 (1.61; 3.10)		1.82 (1.43; 2,31)		1.45 (1.19; 1.75)	
Don't know	(ommited)		(ommited)		0.94 (0.59; 1.49)	
Secondary /higher	1		1			
**Marital status**		** < 0.001**		** < 0.001**		** < 0.001**
Never married	1		1		1	
Married	14.44 (6.77; 30.77)		13.38 (7.11; 25.19)		16.75 (10.31; 27.23)	
Living together	6.64 (2.77; 15.87)		10.02 (5.14; 19.54)		7.96 (4.70; 13.49)	
others	15.15(6.72; 34.15)		12.91 (6.49; 25.70)		12.59 (7.35; 21.58)	
**Wealth index quintile**				** < 0.001**		** < 0.001**
Very poor			1.66 (1.16; 2.37)		1.30 (0.99; 1.72)	
Poor			1.82 (1.28; 2.58)		1.40 (1.06; 1.84)	
Average			2.28 (1.61; 3.24)		1.87 (1.43; 2.43)	
Rich			2.29 (1.62; 3.25)		2.22 (1.70; 2.91)	
Very rich			1		1	

### Estimation of the stillbirth rate

Table 2 depicts the overall and sub-group-specific stillbirth rates for 1998, 2004, and 2011. The crude stillbirths rate from the DHS data indicates a V-shaped evolution: in the first phase, it decreased from 21.9 (95%CI: 18.7–25.1) per 1000 births in 1998 to 16.4 (95%CI: 14.0–18.9) per 1000 births in 2004; in the second phase, it increased slightly up to 17.8 (95%CI: 15.9–19.7) per 1000 births in 2011. This rate tended to increase within age groups (*p* < 0.001).

**Table2:** Multivariate logistic regression

	**1998**		**2004**		**2011**	
**Variables**	**ORa (95% CI)**	**P**	**ORa (95% CI) **^**a**^	**P**	**ORa (95% CI) **^**a**^	**P**
**Age group**		** < 0.001**		** < 0.001**		** < 0.001**
15–19	1		1		1	
20–24	4.23 (1.59; 11.21)		3.69 (1.86; 7.31)		2.31 (1.37; 3.88)	
25–29	6.41 (2.44; 16.85)		5.55 (2.80; 10.98)		2.94 (1.75;4.92)	
30–34	7.86 (2.97; 20.75)		7.33 (3.70; 14.55)		4.67 (2.79; 7.80)	
35–39	11.58 (4.42; 30.30)		9.35 (4.70; 18.57)		6.02 (3.61; 10.04)	
40–44	13.05 (4.94; 34.49)		9.79 (4.88; 19.62)		7.18 (4.28; 12.06)	
45–49	20.66 (7.82; 54.60)		10.91 (5.41; 21.99)		6.89 (4.07; 11.67)	
**Region of residence**				**0.08**		** < 0.001**
Adamaoua			1		1	
Centre			1.56 (0.90; 2.69)		0.58 (0.38; 0.89)	
Douala			0.73 (0.37; 1.44)		0.63 (0.41; 0.98)	
East			2.12 (1.27; 3.53)		0.66 (0.44; 1.00)	
Far-North			1.20 (0.73; 196)		1.09 (0.79; 1.50)	
Littoral			1.69 (0.97; 2.92)		0.63 (0.40; 0.99)	
North			0.31 (0.16; 0.61)		0.77 (0.55; 1.09)	
North-West			0.77 (0.43; 1.39)		0.34 (0.22; 0.53)	
West			1.65 (0.99; 2.74)		0.65 (0.44; 0.96)	
South			1.05 (0.57; 1.92)		0.73 (0.46; 1.15)	
South-West			0.89 (0.48; 1.63)		0.53 (0.34; 0.81)	
Yaoundé			1.34 (0.72; 2.51)		0.72 (0.46; 1.13)	
**Type of residence**		**0.16**		**0.20**		**0.89**
Urban	1		1		1	
Rural	1.22 (0.93; 1.61)		1.21 (0.90; 1.63)		0.98 (0.75; 1.29)	
**Level of education**		**0.56**		**0.40**		**0.70**
No education	1.12 (0.76; 1.65)		1.16 (0.77; 1.73)		0.87 (0.64; 1.18)	
Primary	1.37 (0.97; 1.94)		1.15 (0.87; 1.50)		1.13 (0.90; 1.42)	
Don't know	(ommited)		(ommited)		(ommited)	
Secondary /higher	1		1		1	
**Marital status**		** < 0.001**		**0.002**		** < 0.001**
Never married	1		1		1	
Married	4.19 (1.86; 9.43)		3.55 (1.77; 7.14)		5.66 (3.33; 962)	
Living together	3.11 (1.27; 7.63)		3.12 (1.53; 6.39)		3.94 (2.25; 6.90)	
others	3.84 (1.62; 9.13)		3.12 (1.48; 6.59)		4.04 (2.26; 7.22)	
**Wealth index quintile**				**0.18**		**0.14**
Very poor			1.37 (0.93; 2.01)		1.25 (0.187; 0.14)	
Poor			1.21 (0.78; 1.86)		123 (0.210; 0.24)	
Average			1.43 (0.90; 2.29)		1.49 (0.294; 0.04)	
Rich			1.52 (0.93; 2.49)		1.39 (0.315; 0.15)	
Very rich			1		1	

*ORa* adjusted Odd ratio

In 2004, the regions with the highest reported stillbirth rate included the East, the Littoral, the West, and the Far North, with stillbirth rates of 26.6, 21.5, 21.3, and 20.7 per 1,000 births, respectively. In 2011, the regions with the highest stillbirth rate included the Far North, Douala, Adamawa, and Yaoundé, 23.6, 21.6, 20.4, and 19.6 per 1,000 births, respectively. Women in rural areas had higher stillbirth rates than their counterparts in urban areas (*p* < 0.01), except for the year 2011, when the stillbirth rate was higher in urban than in rural zones (*p* < 0.001).

Concerning the level of education, this rate was higher among women who attended not more than primary school in 1998 (25.3 per 1,000 births; 95%CI: 21.3–29.4) the secondary school in 2004 (17.1 per 1,000 births; 95%CI: 13.5–20.7), and among those who never went to school in 2011 (18.1 per 1,000 births; 95%CI: 14.2–22.0). Single women had the lowest stillbirth rates, especially in the 1998 survey (10.9 per 1,000 births; 95%CI: 3.3–18.5; *p* < 0.001) and in 2011 (10.3 per 1,000 births; 95%CI: 4.8–15.8; *p* < 0.001); separated women had the highest rates in the same periods (Table 2). In 2004, the lowest rate was observed among widows (13.2per 1,000 births; 95%CI: 7.1–19.3), without any significant association between the stillbirth rate and marital status (*p* = 0.106). The very rich had the lowest stillbirth rate in 2011 (15.6 per 1,000 births; 95%CI: 12.2–19.1; Table 2).

### Average Annual Rate of Reduction of stillbirths (AARR), from 1998 to 2011

Rate of change analysis was used to calculate the AARR of stillbirths from 1998 to 2011. Progress in reducing stillbirth rates has been plodding from 1998 to 2011, at a pace of 1.6% per year.

### Factors that may impact stillbirth occurrence

Using logistic regression analysis, we investigated those factors likely influencing stillbirth occurrence in our context, presented in Table 2. Compared to women aged 15–19, others had an increased risk of delivering a stillborn baby (OR 2.4–21.2; *p* < 0.05), except for 2004. In 1998, women living in the North were likely less prone to delivering a stillborn baby (OR 0.3; 95%CI: 0.1–0.5; *p* < 0.001) than women living in the Adamawa region, which was no more the case in 2011. Still, in 2011, illiterate women had statistically significantly higher odds of giving birth to a stillborn baby than women who attended University or College (OR 1.3; 95%CI: 1.0–1.6; *p* = 0.003). Concerning marital status, single women had the highest risk of delivering a stillborn when compared to divorced women (OR 4.1; 95%CI: 1.8–9.2; *p* < 0.001 in 1998 and OR 5.5; 95%CI: 3.2–9.4; *p* < 0.001 in 2011); this finding was not statistically significant in 2004 (Table 2). In 2004 and compared to the very poor women, rich (OR 0.6; 95%CI: 0.4–0.9; *p* = 0.025) and very rich women (OR 0.4; 95%CI: 0.3–0.7; *p* < 0.001) were less prone to giving birth to a stillborn (Table 2).

## Discussion

The crude stillbirth rate in Cameroon was 21.9 per 1,000 births (95%CI: 18.7–25.1) in 1998, 16.4 per 1,000 births (95%CI: 14.0–18.9) in 2004 and 17.8 per 1,000 births (95%CI: 15.9–19.7) in 2011. The stillbirth rate reduction in Cameroon was prolonged from 1998 to 2011, with an AARR of 1.6%. This finding is similar to the 2000 – 2015 AARR for sub-Saharan Africa of 1.4% [2]. A similar observation is found in 2000–2019 AARR in neighboring Nigeria evaluated at 2.9% [12]. The study observed that residing in rural areas, having a low socioeconomic status, advanced maternal age, and low level of education were major factors influencing stillbirth occurrence in our context [11, 13, 14].

Worldwide, the AARR of stillbirths from 2000 – 2015 is estimated at 2% [15]. It has been shown that attainment of the target of 12 per 1,000 births, stillbirths or less by 2030 would require a global AARR of 4·2% from 2015, more than double the present; however, this international projection does not take into account regional variations [15]. Almost 98% of stillbirths occur in low- and middle-income countries, with three-quarters in sub-Saharan Africa and South Asia. Some countries like Bangladesh (AARR 3.4%) and Cambodia (AARR 3.6%) made substantial progress over the past decades. Notwithstanding, there is still a long way to go in sub-Saharan Africa, with the highest stillbirth rates [15]. Special efforts should be made in countries of his region like Cameroon to reduce the burden of stillbirths.

Five priority strategic objectives have been defined to accelerate stillbirth reduction: (i) strengthen and invest in care around the time of birth, with a particular focus on improving the quality and experience of care; (ii) strengthen health systems to optimize the organization and delivery of care, the workforce, commodities, and innovation; (iii) reach every woman and newborn by minimizing inequities in access to and coverage of care; (iv) harness the power of parents, families, and communities, and engage with society, and (v) improve data for decision making and accountability by establishing national registration and vital statistics systems [15]. In order to achieve these targets, an essential package of effective interventions should be designed and implemented to reach every woman and pregnancy. The choice and prioritization of interventions should be tailored to each country depending on local realities regarding causes and/or risk factors for stillbirth [15].

Accordingly, we found that women with a low socioeconomic status, a low level of education, and/or living in rural areas were more at risk of experiencing a stillbirth, thus corroborating findings [3]. In 2011, the regions with the highest stillbirth rates were the Far North, Douala, Adamawa, and Yaoundé, with 23.6, 21.6, 20.4, and 19.6 per 1,000 births. We believe that to accelerate stillbirth reduction in our country, it would be essential to focus on these priority regions with the highest stillbirth rates. Furthermore, there is a need to increase the number of births in health facilities and improve the quality of care provided to women during childbirth. Health facility deliveries did not improve enough and were 58% in 1998, 63% in 2011, and 61.4% in 2014, according to DHS 1998, DHS-MICS 2011, and MICS 2014. Previous studies have estimated that optimal quality of care around childbirth could avert 531 000 stillbirths globally by 2020 [15]. Moreover, women empowerment and education should be emphasized, especially in these priority regions, as wealth and a high education level protect against the odds of having a stillborn child [15].

However, this study needs to be interpreted with some limitations. First, the DHS collected only information relating to the last pregnancy, which ended in a miscarriage, and abortion or stillbirth, making the completeness of information gathered uncertain. Second, in 2018 the DHS data collected did not permit us to determine the crude stillbirth rate; therefore, the trend could not include that value. Also, data were not disaggregated enough to explore the potential risk factors described in the literature. Hence, the subsequent DHS questionnaire should be improved to better capture the stillbirth burden information and refine the analysis of explanatory factors [11]. To the best of our knowledge, despite these limitations, this is the first comprehensive study summarizing the epidemiology of stillbirths in Cameroon from 1998 to 2011 based on a nationally representative survey, which has the advantage of assessing the actual burden of stillbirths.

## Conclusion

The stillbirth rate is an excellent indicator of the quality and equity of health care. This study estimated the stillbirth rates in 1998, 2004, and 2011, with an average annual reduction of 1.6 between 1998 and 2011. Women residing in rural areas, those with a low socioeconomic status, advanced maternal age, or a low level of education, had an increased risk of experiencing a stillbirth. These main risk factors call for the urgent need to implement targeted strategies like improving the quality of care around childbirth, empowering and educating women, and prioritizing regions with the heaviest burden. Furthermore, better data availability shall accurately indicate Cameroon's actual burden of stillbirths. 

## Data Availability

The data that support the findings of this study are available from Cameroon National Institute of Statistics but restrictions apply to the availability of these data, which were used under license for the current study, and so are not publicly available. Data are however available from the authors upon reasonable request and with permission of the Director of this institute.

## Abbreviations

AARR: Average Annual Rate of Reduction
CI: Confidence Interval
DHS: Demographic and Health Survey
ICD-10: International Classification of Diseases
MICS: Multi-Index Cluster Survey
ORa: Adjusted Odd Ratio
OR: Odds Ratio
